# Current practice of thoracic anaesthesia in Europe – a survey by the European Society of Anaesthesiology Part I – airway management and regional anaesthesia techniques

**DOI:** 10.1186/s12871-021-01480-w

**Published:** 2021-11-01

**Authors:** Jerome Defosse, Mark Schieren, Torsten Loop, Vera von Dossow, Frank Wappler, Marcelo Gama de Abreu, Mark Ulrich Gerbershagen

**Affiliations:** 1 Department of Anaesthesiology and Intensive Care Medicine, University Witten/Herdecke, Medical centre Cologne-Merheim, Cologne, Germany; 2 Department of Anesthesiology and Critical Care, Medical Center-University of Freiburg, Faculty of Medicine, University of Freiburg, Freiburg, Germany; 3 Institute of Anesthesiology, Heart and Diabetes Center North Rhine Westphalia, Bad Oeynhausen, Germany; 4 Department of Anaesthesiology and Intensive Care Medicine, Pulmonary Engineering Group, Technische Universität Dresden, University Hospital Carl Gustav Carus, Dresden, Germany; 5 Department of Intensive Care and Resuscitation, Cleveland Clinic, Anesthesiology Institute, Ohio, USA; 6 Department of Outcomes Research, Cleveland Clinic, Anesthesiology Institute, Ohio, USA; 7 Department of Anaesthesiology, University Witten/Herdecke, Hospital Holweide, Cologne, Germany

**Keywords:** Thoracic anaesthesia, One-lung ventilation, Bronchial blocker, Regional anaesthesia, Thoracic surgery

## Abstract

**Background:**

The scientific working group for “Anaesthesia in thoracic surgery” of the German Society of Anaesthesiology and Intensive Care Medicine (DGAI) has performed an online survey to assess the current standards of care and structural properties of anaesthesia workstations in thoracic surgery.

**Methods:**

All members of the European Society of Anaesthesiology (ESA) were invited to participate in the study.

**Results:**

Thoracic anaesthesia was most commonly performed by specialists/board-certified anaesthetists and/or senior/attending physicians. Across Europe, the double lumen tube (DLT) was most commonly chosen as the primary device for lung separation (461/ 97.3%). Bronchial blockers were chosen less frequently (9/ 1.9%).

Throughout Europe, bronchoscopy was not consistently used to confirm correct double lumen tube positioning. Respondents from Eastern Europe (32/ 57.1%) frequently stated that there were not enough bronchoscopes available for every intrathoracic operation. A specific algorithm for difficult airway management in thoracic anaesthesia was available to only 18.6% (*n* = 88) of the respondents. Thoracic epidural analgesia (TEA) is the most commonly used form of regional analgesia for thoracic surgery in Europe. Ultrasonography was widely available 93,8% (*n* = 412) throughout Europe and was predominantly used for central line placement and lung diagnostics.

**Conclusions:**

While certain „gold standards “are widely met, there are also aspects of care requiring substantial improvement in thoracic anaesthesia throughout Europe.

Our data suggest that algorithms and standard operating procedures for difficult airway management in thoracic anaesthesia need to be established. A European recommendation for the basic requirements of an anaesthesia workstation for thoracic anaesthesia is expedient and desirable, to improve structural quality and patient safety.

**Supplementary Information:**

The online version contains supplementary material available at 10.1186/s12871-021-01480-w.

## Background

The anaesthetic management of patients undergoing thoracic surgery may be challenging. The need for lung separation, one-lung ventilation and bronchoscopy, as well as the frequent need for intervention by the anaesthesiologist in the context of hypoxia, e.g. due to DLT dislocation, increase the complexity of airway management.

Despite of the availability of a large variety of airway and lung separation devices, little is known about their use throughout Europe. Furthermore, fundamental structures of care in thoracic surgery and anaesthesia, such as perioperative patient pathways, provider qualifications as well as training and educational programmes are unknown. Although some structures of care have been evaluated in select areas, such as United Kingdom [1], the middle east [2], Italy [3] and Germany [4], Europe-wide information is not available.

After conducting a similar study in Germany [4], the scientific working group for “Anaesthesia in thoracic surgery” of the German Society of Anaesthesiology and Intensive Care Medicine (DGAI) has performed an online survey to assess the current standards of care and structural properties of anaesthesia workstations in thoracic surgery in Europe.

## Methods

We conducted an observational cross-sectional study without any interventions. Data was collected using an online questionnaire consisting of 5 sections and a total of 45 items. All members (20,000) of the European Society of Anaesthesiology (ESA) were invited to participate in the study via email (12/09/2017) and social media (i.e. the official ESA Facebook site (11/10/2017). The online questionnaire could be accessed and completed from September 12th to October 31st 2017 using the survey tool LimeSurvey®.

The survey’s first section assessed basic information of the participants, such as ESA membership status, country of practice, as well as structural characteristics of their hospital and department of anaesthesia. Only participants working in hospitals that performed at least 1 thoracic operation per month were permitted to complete the rest of the survey.

The second section investigated the primary method of airway management, when one-lung ventilation is required, as well as the management of expected and unexpected difficult airways in thoracic anaesthesia.

The survey’s third and fourth section focused on intraoperative ventilator settings during one-lung ventilation and troubleshooting in case of impaired gas exchange.

The fifth and final section targeted the use of regional anaesthetic techniques and ultrasonography.

To be eligible for inclusion, respondents were required to complete at least the first two sections of the study. We excluded respondents, who were practicing outside of Europe or whose hospital did not perform at least 1 intrathoracic operation per month.

For purposes of data analysis and presentation, the survey sections were grouped according to their content. This study presents the results of the survey sections 1, 2 and 5, which predominantly look at the technical aspects of thoracic anaesthesia. Sections 3 and 4, dealing with ventilation and oxygenation will be published separately.

All methods were carried out in accordance with the guidelines and regulations of the European Society of Anaesthesiology and has been performed in accordance with the Declaration of Helsinki. Our pertinent local IRB (Institutional review board of Medical Centre Cologne-Merheim (MMC-IRB)) approved the survey and waived the requirement to obtain informed consent because only ESA members were invited to participate anonymously and participants were assumed to be adults and legally competent.

To investigate regional differences throughout Europe, participants of different nations were clustered according to the „Standard country or area codes for statistical use (M49) “of the United Nation Statistics Division (UNSD) into four main regions: northern (NE), eastern (EE), southern (SE) and western Europe (WE) (Supplement 1) (https://unstats.un.org/unsd/methodology/m49/).

Descriptive statistical analysis was performed using SPSS Statistics® (Version 25.0, IBM® Corp., Armonk, USA) and Microsoft-Excel® 2016 (Microsoft Corp., Redmond, USA). Descriptive data are presented as absolute and relative frequencies (n / %). Unless stated otherwise, the relative values refer to the total number of respondents of either the entire study (*n* = 474) or the specified region (NE: *n* = 55; EE: *n* = 57; SE: *n* = 105; WE: *n* = 257). The chi-squared test was chosen for comparisons of categorical variables. A *p*-value ≤0.05 was considered statistically significant.

## Results

A total of 752 ESA members accessed the survey. Five hundred fifty-four respondents were eligible for inclusion. After exclusion of 44 respondents, whose hospitals did not perform thoracic surgery and 36 respondents, who were practicing outside of Europe, 474 completed surveys were included in the analysis.

### Section 1: general information and hospital characteristics

The 474 included respondents were practicing in 33 different European countries (Table 1; Supplement 1). The majority of respondents were from Western Europe (257/ 54,2%) and in particular from Germany (124/ 26,2%).

**Table 1:** Regional distribution of respondents throughout Europe

	n	%
Northern Europe	55	11.6%
Eastern Europe	57	12%
Southern Europe	105	22.2%
Western Europe	257	54.2%

Regarding the professional status, most respondents had completed anaesthesia specialty training (Specialist/Certified Anaesthesiologist: 175/ 36,9%) or were occupying senior/supervising positions (Consultant/Attending/Senior Physician: 175/ 36,9%) (Table 2). Trainees (Trainee/Registrar/ Junior Physician: 59/12,4%) and department heads (head of department: 50/10,5%) participated less frequently.

**Table 2:** Basic characteristics of survey respondents

		Northern Europe *n* = 55	Eastern Europe *n* = 57	Southern Europe *n* = 105	Western Europe *n* = 257	Total
						*n* = 474
For how many years have you been working in the field of Anaesthesiology?	< 3 years	5.5%	5.3%	6.7%	7.0%	6.5%
	4–6 years	14.5%	17.5%	19.0%	14.4%	15.8%
	7–9 years	9.1%	21.1%	13.3%	8.9%	11.4%
	10–19 years	40.0%	24.6%	30.5%	33.1%	32.3%
	> 20 years	30.9%	31.6%	30.5%	36.6%	34.0%
What is your hospital’s level of care?	University hospital	76.4%	57.9%	61.0%	42.8%	52.5%
	Maximum care	14.5%	21.1%	24.8%	29.6%	25.7%
	Extended care	7.3%	12.3%	11.4%	22.6%	17.1%
	Basic care	0.0%	1.8%	2.9%	3.1%	2.5%
	Specialized clinic for thoracic surgery	1.8%	7.0%	0.0%	1.9%	2.1%
How many intrathoracic (non-cardiosurgical) operations are performed at your hospital per month?	1–5/month	14.5%	26.3%	18.1%	14.0%	16.5%
	6–10/month	9.1%	8.8%	24.8%	20.2%	18.6%
	11–50/month	54.5%	38.6%	40.0%	49.4%	46.6%
	> 50/month	21.8%	26.3%	17.1%	16.3%	18.4%

Overall, most participants were experienced anaesthetists (≥10 years of experience: 314/ 66,2%) and working in hospitals with a high level of care (university hospital: 249/52,5%; hospital with maximum level of care: 122/25,7%) (Table 2). These facilities were often large medical centres (≥800 beds: 221/ 46,6%) and performed thoracic operations on a routine basis (> 11 thoracic operations/month: 309/ 65,2%).

Regarding the qualifications of surgeons, most thoracic operations were performed by specialized thoracic surgeons (NE: 51/ 92.7%; EE: 45/ 78.9%; SE: 92/ 87.6%; WE: 180/ 25.7%) and/or general surgeons certified for thoracic surgery (NE: 6/ 10.9%; EE: 14/ 24.6%; SE: 9/ 8.6%; WE: 104/ 40.5%). Throughout Europe, only the minority of respondents (32/ 6.8%) stated that general surgeons, who were not specifically certified for thoracic surgery were performing thoracic operations.

Pneumonectomies were most commonly marked the most invasive intrathoracic surgical procedure performed at the respondents‘ hospitals (NE: 17/ 30.9%; EE: 29/ 50.9%; SE: 51/ 48.6%; WE: 88/ 34.2%). Compared with the remaining regions, respondents’ hospitals from Northern Europe more frequently performed lung transplantations (NE: 11/ 20%; EE: 3/ 5.3%; SE: 7/ 6.7%; WE: 37/ 14.4%).

Thoracic anaesthesia was most commonly performed by specialists/board-certified anaesthetists (317/ 66.9%) and/or senior/attending physicians (265/ 55.9%). Unsupervised trainees/registrars rarely performed thoracic anaesthesia (5/ 1.1%). These results were comparable in all regions. There were marked regional differences with regard to the number of respondents that stated that supervised trainees/residents were performing thoracic anaesthesia (NE: 16/ 29.1%; EE: 19/ 33.3%; SE: 7/ 6.7%; WE: 146/ 56.8%) (*p* = 0.000).

Training and education in thoracic anaesthesia was comparable across the regions and was most commonly conducted during specific in-house rotations (352/ 74.3%). External training rotations were less common (80/ 16.9%). Overall, the duration of training rotations varied widely with an average of 4.1 months (*n* = 270) for in-house rotations and 7.2 months (*n* = 71) for external rotations.

With regard to treatment units chosen for postoperative care, there were regional differences throughout Europe. Multiple answers were possible. Post anaesthesia recovery rooms were more frequently used in Northern (35/63.6%) and Western Europe (163/ 63.4%) than in Southern (51/ 48.6%) or Eastern Europe (23/ 40.4%) (*p* = 0.002). Only respondents from Western Europe chose intermediate care units on a more regular basis (107/ 41.6%). With an average response rate of 23.5% (*n* = 51), the remaining European regions used intermediate care units less frequently. An immediate postoperative transfer to intensive care units was more common in Eastern (43/ 75.4%) and Western Europe (180/ 70%), than in Southern (55/ 52.4%) and Northern Europe (23/ 41.8%).

### Section 2: airway management for lung separation

All across Europe, the DLT was most commonly chosen as the primary device for lung separation (461/ 97.3%). Regarding the level of experience with DLT, the majority of respondents were regular (149/ 31.4%) or expert users (229/ 48.3%). No regional differences were noted (*p* = 0.77).

Bronchial blockers were rarely chosen as the primary device (9/ 1.9%). The level of experience with the use of bronchial blockers was markedly lower compared to double lumen tubes and demonstrated more regional variations (Table 3). We did not distinguish between the different products in the survey, e.g. the Univent tube was subsumed under bronchial blockers.

**Table 3:** Level of experience with bronchial blockers

	Northern Europe *n* = 55	Eastern Europe *n* = 57	Southern Europe *n* = 105	Western Europe n = 257	Total *n* = 474
No experience	32.7%	49.1%	24.8%	19.5%	25.7%
Infrequent use, supervision required	20.0%	19.3%	20.0%	23.3%	21.7%
Occasional use, no supervision required	30.9%	15.8%	26.7%	29.2%	27.2%
Regular use	10.9%	7.0%	16.2%	14.4%	13.5%
expert	5.5%	8.8%	12.4%	13.6%	11.9%

Bronchoscopic control of correct tube positioning is not consistently used throughout Europe. While respondents from Northern (45/ 81.8%) and Western Europe (211/ 82.1%) routinely used bronchoscopy for airway positioning, this was less frequently the case in Southern (60/ 57.1%) and particularly in Eastern Europe (12/ 21.1%) (*p* = 0.000). In case of right-sided double lumen tube placement, bronchoscopy was used routinely by 28.1% (*n* = 16) of Eastern European respondents.

Respondents from Eastern Europe (32/ 57.1%) frequently stated that there are not enough bronchoscopes available for every intrathoracic operation. This was less commonly the case in the other regions (NE: 4/ 7.5%; SE: 32/ 31.1%; WE: 16/ 6.3%) (467 respondents).

The majority of respondents confirmed that a general difficult airway algorithm was used in their departments (338/ 71.3%). A specific algorithm for difficult airway management in thoracic anaesthesia was available to 18.6% (*n* = 88) of the respondents.

**Fig. 1:**
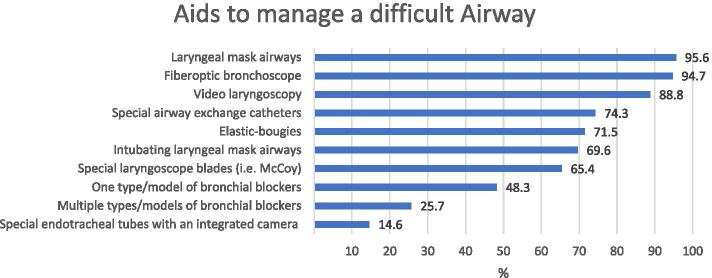
Availability of different aids and devices for difficult airway management in thoracic anaesthesia (474 respondents)

The availability of different aids and devices used for the management of difficult airway in thoracic anaesthesia are displayed in Fig. 1. Bronchial blockers were generally available to 71,9% (*n* = 341) of the respondents.

**Fig. 2:**
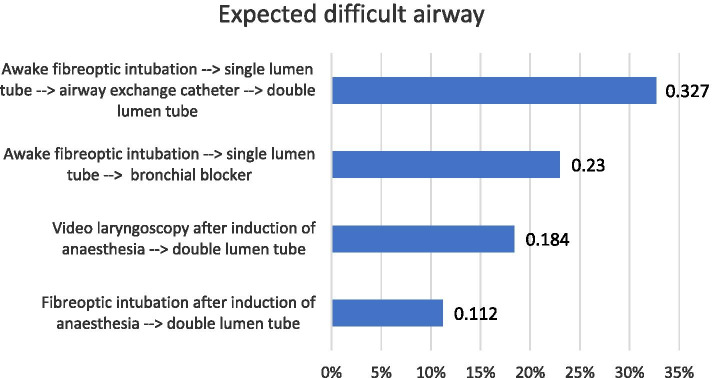
Primary strategy for management of an expected difficult airway in thoracic anaesthesia (474 respondents)

**Fig. 3:**
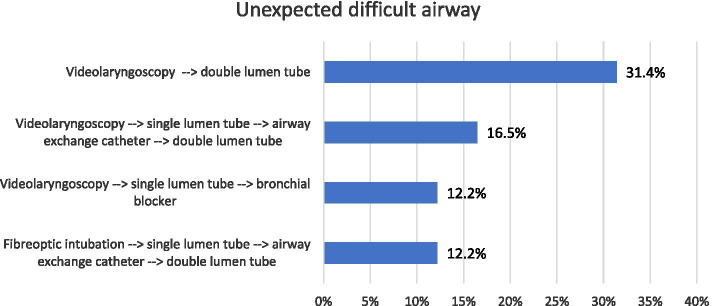
Primary strategy for management of an unexpected difficult airway in thoracic anaesthesia (474 respondents)

Primarily chosen strategies for the management of expected and unexpected difficult airways in thoracic anaesthesia are displayed in Figs. 2 and 3.

### Section 5: pain management and ultrasound use

Throughout Europe, epidural catheters were most frequently used for perioperative pain management in thoracic anaesthesia (Table 4).

**Table 4:** Specific techniques used for perioperative pain management in thoracic anaesthesia. Multiple answers were possible (439 respondents)

	Northern Europe *n* = 51	Eastern Europe n = 51	Southern Europe *n* = 97	Western Europe *n* = 240	Total *n* = 439
Thoracic epidural analgesia	72.5%	64.7%	79.4%	90.8%	83.1%
Paravertebral blockade (single shot)	17.6%	17.6%	16.5%	10.8%	13.7%
Paravertebral blockade (catheter)	25.5%	25.5%	15.5%	10.8%	15.3%
i.v. PCA	27.5%	23.5%	22.7%	25.0%	24.6%

Based on the answers of 439 respondents, ultrasonography was widely available in thoracic anaesthesia (412/ 93.8%). They were predominantly used for the placement of central lines (92%), lung diagnostics (66.1%), arterial puncture and catheterization (53.3%), and less frequently for paravertebral blockades (26.2%) or the placement of epidural catheters (4.3%) (439 respondents).

## Discussion

This Europe-wide survey yields multiple important insights and regional differences with regard to the structures of care in thoracic anaesthesia.

Especially in comparison to Western Europe, there was a high number of Northern European respondents working in university hospitals (76.4%). Based on these results, one might speculate that in contrast to Western Europe, Northern European nations perform intrathoracic operations predominantly at large specialized university centres. This hypothesis could be further supported by the high rate of specialized thoracic surgeons and the number of respondents performing lung transplantations in Northern Europe. In contrast, general surgeons certified for thoracic surgery are commonly performing intrathoracic operations in Western Europe (40.5%). It would be interesting, to investigate the impact of specialization of care in thoracic centres with highly qualified personnel and high case numbers on patient-centred outcomes, such as morbidity and mortality.

The DLT was most commonly chosen for lung separation and there was a high level of expertise. Considering that DLT intubations may be difficult in 2.8% of cases, a structured approach to difficult airway management of a DLT in thoracic anaesthesia is necessary [5]. According to our results, this was rarely the case. While regular difficult airway algorithms are widely available and useful to ensure adequate oxygenation, in case of thoracic anaesthesia the frequent need for lung separation and one-lung ventilation needs to be taken into account. In thoracic anaesthesia, in addition to the establishment of a safe airway for oxygenation of the patient, there is also the need for lung separation and one-lung ventilation. The three areas of securing the airway, lung separation and one-lung ventilation are so complex that, in the authors’ view, a detailed European recommendation would be necessary, as our survey showed a very heterogeneous approach in Europe. In this survey, we focused mainly on the airway and did not look in detail at the issue of one-lung ventilation.

The maintenance of spontaneous breathing is the current standard of care for the management of an expected difficult airway in difficult conditions in different European recommendations. Awake intubation under spontaneous breathing using DLT is certainly challenging due to the diameter of the DLT and can only be performed in the minority of patients. Endoscopic awake intubation using a single lumen tube is technically simpler and therefore more likely to be successful. When lung separation is required, the single lumen tube may either be exchanged to a DLT or be equipped with a bronchial blocker. The use of airway exchange catheters to change from a single lumen to a DLT has been reported to have a failure rate of 39.9% [6]. The risks of potentially losing an established airway in difficult airway conditions should be carefully considered. For this reason, the use of a bronchus blocker at this point could be a safe alternative for lung separation in case of a failed intubation using DLT. By using a bronchial blocker, there is no need to jeopardise an airway that has already been secured by a single lumen tube. In theory, this appears to be a safer option, although there is no evidence to support this claim. Despite the advantageous safety profile, there are also downsides to the use of bronchial blockers. Lung deflation is not as fast and effective, which could worsen the conditions for the surgeon. Furthermore, bronchial blockers are generally unsuitable for surgical procedures involving the ipsilateral mainstem bronchus (e.g. sleeve resections). According to our results, however, only 71.9% of respondents had access to bronchial blockers in their department. Furthermore, most providers had no or limited experience with the use of bronchial blockers and required direct supervision. In case of a difficult airway and the urgent need for lung separation, this lack of availability and expertise, even in specialised centres, needs to be viewed critically. Despite increased cost and higher rates of dislocation, bronchial blockers should be used regularly in simulation exercises and elective intrathoracic surgery to increase the level of experience and patient safety. It has been shown that 6 bronchial blockers placements are enough to significantly improve provider dexterity. However, 15 uses are required to acquire adequate skills for the correction of mispositioned blockers [7]. This emphasises the need for regular hands-on training and bronchial blocker use to acquire and maintain the necessary skills. There is also no evidence comparing individual techniques for lung separation in the management of a known difficult airway. In light of the wide spectrum of available approaches to difficult airway management and lung separation in thoracic anaesthesia, a general recommendation applicable to all clinical scenarios is not possible. Decisions must be made on an individual patient basis, taking patient related factors, surgical requirements as well as local availability and provider experience into account.

Video laryngoscopy is an invaluably helpful tool in the management of difficult airways in non-thoracic anaesthesia. There are conflicting results, however, regarding the utility of video laryngoscopy for the placement of double lumen tubes. While one study found higher success rates, a shorter duration of intubation and a lower incidence of postoperative hoarseness with the use of the hyperangulated Glidescope® for the video laryngoscopy [8], another study reported mostly opposite results for the same device [9]. Especially for less experienced providers, the use of the relatively large hyperangulated video laryngoscopy blade in combination with inflexible and thick double lumen tubes made tracheal intubation more difficult [9]. Since the introduction of videolaryngoscopy and its well proven utility in single lumen tube intubation, many different variants (e.g. Macintosh vs. hyperangulated blade) are available in Europe. A randomised comparison of the different shapes and sizes available in relation to the intubation of a DLT would certainly be helpful in the future.

With regard to ensuring lung separation, the regional heterogeneity with regard to the availability and use of bronchoscopy in thoracic anaesthesia throughout Europe is remarkable. Visual confirmation of correct double lumen tube position is considered as gold standard, as it has been repeatedly shown to be superior to auscultation and clinical exam findings [10, 11]. Especially in Eastern Europe, the majority of respondents (57%) did not have access to a bronchoscope for every intrathoracic operation. A European recommendation including the constant availability of bronchoscopy in thoracic anaesthesia, may be helpful in budget discussions with hospital administrators. While being expensive, bronchoscopy is an essential tool for the placement of double lumen tubes and bronchial blockers and has an immediate impact on patient safety.

Thoracic epidural analgesia (TEA) was the preferred perioperative method of pain management during thoracic surgery and was used much more commonly than paravertebral blockades (PVB). Our findings regarding the use of regional anaesthetic techniques (TEA: 83.1%; PVB single shot: 13.7%, PVB catheter: 15.3%) are comparable to similar investigations performed in the United Kingdom (TEA: 62%, PVB: 30%) [1] and Germany (TEA: 84.5%; PVB (single shot): 8.6%; PVB (catheter): 8%) [4]. Paravertebral blocks are a suitable alternative to TEA, as the efficacy regarding analgesic quality is comparable [1, 12, 13], yet, the incidences of perioperative hypotension and urine retention are lower [12]. Considering the current trend towards less invasive intrathoracic operating techniques (i.e. VATS), the indications for TEA may be decreasing. In this regard, especially ultrasound guided paravertebral blockade appear advantageous, given the ease of use and preferable side effects profile [14]. Since a variety of new ultrasound-guided regional anaesthesia techniques are emerging in the field of thoracic anaesthesia (e.g., serratus anterior or erector spinae blocks), a detailed investigation of the current practice of regional anaesthesia in thoracic surgery in Europe would be desirable. The widespread availability of ultrasound equipment in Europe is promising with regard to perioperative patient safety.

Certain limitations may apply to our findings. It is not possible to determine a response rate, as the invitation to participate was distributed via email and social media. The number of completed questionnaires (*n* = 474), however, is comparable to previous ESA surveys [15]. Colleagues with a particular interest in thoracic surgery may have been more inclined to participate in our survey. The impact of this risk of bias, however, is unclear. Regionally, the number of respondents were not equally distributed throughout Europe. Western European practitioners were overrepresented (257/ 54.2%), with high number of German respondents (124/ 26,2%). This may limit the generalisability of our findings. It is unclear, whether this differs from the regional distribution of ESA members in general. Regarding certain survey topics, more detailed questions would have been useful, e.g. different variants of SGA were not differentiated and a “nonintubated” approach was not addressed.

## Conclusions

The gold standard of bronchoscopic control of the correct position of DLT or of bronchus blocker cannot be met in many areas of Europe due to lack of bronchoscopic equipment. More than 50% of the participants in this survey are either unable to place a bronchus blocker at all or require supervision. There is significant heterogeneity throughout Europe regarding anaesthetic management in case the primarily chosen method for lung separation fails. A standardized approach to difficult airway management is missing. There is a lack of uniform European recommendations regarding the establishment of a lung separation and one-lung ventilation in difficult situations. In this context, the availability of bronchus blockers and provider expertise need to increase in order to improve patient safety. While certain „gold standards “of care, such as the use of ultrasonography and regional analgesia techniques, are widely met throughout Europe, there are also aspects requiring substantial improvement.

## Supplementary Information

**Additional file 1.**

## Data Availability

The datasets used and/or analysed during the current study are available from the corresponding author on reasonable request.

## Abbreviations

DGAI: German Society of Anaesthesiology and Intensive Care Medicine
ESA: European Society of Anaesthesiology
UNSD: United Nation Statistics Division
NE: Northern Europe
EE: Eastern Europe
SE: Southern Europe
WE: Western Europe
TEA: Thoracic epidural anaesthesia
PVB: Paravertebral blockades
VATS: Video-assisted thoracoscopic surgery
